# Evolocumab Reduces Oxidative Stress and Lipid Peroxidation in Obese Zucker Rats

**DOI:** 10.3390/pathophysiology32010005

**Published:** 2025-01-21

**Authors:** Martina Cebova, Radoslava Bulkova, Olga Pechanova

**Affiliations:** 1 Institute of Normal and Pathological Physiology, Center of Experimental Medicine, Slovak Academy of Sciences, 813 71 Bratislava, Slovakia; martina.cebova@savba.sk (M.C.); radka.rehakova@gmail.com (R.B.); 2 Institute of Pathophysiology, Faculty of Medicine, Comenius University, 811 08 Bratislava, Slovakia

**Keywords:** PCSK9, evolocumab, hyperlipidemia, LDL cholesterol, oxidative stress, lipid peroxidation, NADPH oxidase, NF-kappaB, nitric oxide

## Abstract

**Background/Objectives**: Evolocumab inhibits proprotein convertase subtilisin/kexin type 9 (PCSK9) binding to low-density lipoprotein (LDL) receptors, thus allowing more LDL receptors to remove LDL cholesterol from the blood. We aimed to determine the effects of evolocumab on the plasma lipid profile, reactive oxygen species (ROS), and nitric oxide (NO) generation in the heart of adult male obese Zucker rats. **Methods**: The rats were divided into lean and obese controls and obese rats treated with evolocumab subcutaneously at a dose of 10 mg/kg every two weeks. After 6 weeks, the lipid profile was determined in the plasma, and NO synthase (NOS) activity, thiobarbituric acid reactive substance (TBARS), conjugated diene (CD) concentration, and protein expression of nicotinamide adenine dinucleotide phosphate (NADPH) oxidase, nuclear factor kappaB (NF-κB), endothelial NOS (eNOS), and phosphorylated eNOS (peNOS) were measured in the heart. **Results**: Evolocumab treatment did not reduce body weight, relative heart weight, or systolic blood pressure in obese Zucker rats. Evolocumab treatment, however, reduced plasma LDL levels, TBARS, and CD concentrations along with decreasing expression of NADPH oxidase and NF-kappaB proteins in the heart. On the other hand, evolocumab had no effect on NOS activity or eNOS and peNOS protein expression. **Conclusions**: Besides its lipid-lowering effect, evolocumab may exert antioxidant properties and protect cardiomyocytes from lipid peroxidation while not affecting NO production.

## 1. Introduction

Cardiovascular diseases represent the most common cause of morbidity and mortality worldwide. Although the causes of cardiovascular diseases are multifactorial, disorders in lipid metabolism are among the most common risk factors for myocardial infarction, ischemic heart disease, or stroke [1]. Depending on the abnormally increased lipid in the blood, hypertriglyceridemia, hypercholesterolemia, or their combination–hyperlipidemia are distinguished. Inhibitors of 3-hydroxy-3-methylglutaryl coenzyme A (HMG-CoA) reductase–statins have a dominant position in the treatment of hyperlipidemia [2,3]. The pleiotropic effects of statins include inhibition of cellular cholesterol, increased expression of low-density lipoprotein (LDL) receptors [4], and improvement of endothelial function through the activation of nitric oxide synthase (NOS) and decreased production of reactive oxygen species (ROS) [5,6]. However, the administration of statins suppresses the formation of coenzyme Q10 (CoQ10), which is produced via the same biosynthetic pathway as cholesterol [6,7,8]. Although statins are quite well tolerated, their side effects are dose-dependent. In addition, some patients are intolerant to statin therapy and develop cardiovascular diseases despite the maximum dose [9].

Proprotein convertase subtilisin/kexin type 9 (PCSK9) inhibitors may represent a new strategy in lipid-lowering therapy without statin-like side effects [3,10]. PCSK9 is a secretory protease, initially produced as an inactive zymogen. Its activation requires intramolecular autocatalytic cleavage within the endoplasmic reticulum of hepatic cells. After leaving the endoplasmic reticulum, PCSK9 undergoes further processing in the Golgi apparatus before entering the circulation. Once in circulation, PCSK9 inhibits the recycling of LDL receptors to the cell surface, thereby reducing the cellular uptake of plasma LDL [10,11]. The expression of PCSK9 is regulated by sterol regulatory element-binding protein 2 (SREBP-2), which enhances LDL receptor degradation and contributes to elevated plasma LDL levels [12,13]. Beyond lipid metabolism, PCSK9 may interact with Toll-like receptors (TLRs) in macrophages, activating the nuclear factor kappa-B (NF-κB) pathway and promoting NF-κB translocation to the nucleus. This activity can influence the inflammatory microenvironment within the myocardium and vessel wall [14,15]. Knowing the PCSK9 pathway, this protease has become an important LDL-lowering target.

PCSK9 inhibitors prevent the binding of PCSK9 to LDL receptors, thereby inhibiting their degradation and enhancing LDL receptor availability [10,13]. Several strategies have been developed to target PCSK9 inhibition, utilizing various mechanisms, including monoclonal antibodies, synthetic small interfering RNA (siRNA) against PCSK9, vaccination, and small molecules [3]. Among human monoclonal antibodies, evolocumab and alirocumab have been approved by the FDA and are currently marketed. In addition to their ability to lower LDL cholesterol levels, these inhibitors may also improve the lipid profile by increasing high-density lipoprotein (HDL) cholesterol and reducing total cholesterol and lipoprotein(a) levels, ultimately leading to a reduction in plaque volume [16,17]. It is well established that PCSK9 monoclonal antibodies exhibit superior efficacy compared to long-term statin therapy, statin–ezetimibe combination therapy, or ezetimibe monotherapy [18].

Preclinical studies of PCSK9 antibodies showed that these effectively bind to the EGF-binding domain and reduce LDL cholesterol (LDL-c) in C57BL/6 mice on a fat-rich diet and cynomolgus monkeys [13,19]. According to recent human studies, monoclonal antibodies have the potential to be used as an alternative to statins [20].

Although evolocumab is a human monoclonal immunoglobulin G2 (IgG2), its antioxidant and anti-inflammatory effects were demonstrated in Sprague–Dawley rats [21] and transgenic rats [22]. Thus, the aim of our study was to determine the effects of evolocumab on the plasma lipid profile, reactive oxygen species (ROS), and nitric oxide (NO) generation in the hearts of adult male obese Zucker rats.

The outbred obese Zucker rat, characterized mainly by hyperlipidemia, is the best-known and most recommended model of genetic obesity. This model is concurrently well established in searching for the causes of cardiovascular complications in metabolic syndrome [23,24]. Obese Zucker rats have been successfully used in the study of coronary heart disease or heart failure, especially in the search for the intersections of the pathomechanisms of these diseases with obesity and metabolic syndrome pathways [23,25]. Since hyperlipidemia, especially elevated LDL cholesterol, is considered one of the causes of endothelial dysfunction, which is associated with atherosclerosis and cardiovascular complications [5,26], our aim was to study the effects of evolocumab on the disturbances that most contribute to endothelial dysfunction and myocardial ischemia, namely the excessive production of ROS and reduced generation of endothelial nitric oxide. If evolocumab has a positive effect on any of these disorders, it could also be effective in treating obesity-related heart disease, the most serious complication of human obesity.

## 2. Materials and Methods

### 2.1. Chemicals

The majority of chemicals and reagents were procured from Sigma-Aldrich (Saint-Louis, MO, USA). For reagents sourced from other suppliers, the respective company is specified. Evolocumab was supplied by Amgen Technology (Dublin, Ireland) Unlimited Company.

### 2.2. Animals and Treatment

All experimental procedures and protocols were approved by the Ethics Committee of the Center for Experimental Medicine, Institute of Normal and Pathological Physiology, Slovak Academy of Sciences, and the State Veterinary and Food Administration of the Slovak Republic (Ro-1998/15-221). They are in accordance with the European Convention for the Protection of Vertebrate Animals Used for Experimental and Other Scientific Purposes and Directive 2010/63/EU of the European Parliament and fully comply with the ARRIVE guidelines.

Twelve-week-old male obese Zucker (fa-/fa-) rats were procured from Charles River Laboratories (Wilmington, MA, USA). The animals were housed in pairs under controlled environmental conditions, including a 12 h light/dark cycle, constant humidity levels (45–65%), and a stable temperature range of 20–22 °C. The rats were divided into lean and obese controls and obese rats administered evolocumab subcutaneously every two weeks at a dose of 10 mg/kg. The treatment dose was selected according to the weight-based equivalent of the human dose [27] and the faster metabolism of rats. The treatment lasted for 6 weeks. Each experimental group included six animals, all of which were maintained on a standard diet provided ad libitum. Individual daily water intake was monitored and adjusted as required to ensure proper hydration.

### 2.3. Blood Pressure and Weight Parameters

Systolic blood pressure (SBP) was assessed weekly using a non-invasive tail-cuff plethysmographic method, with each measurement performed in triplicate to ensure accuracy. Upon completion of the treatment period, the animals were euthanized, and their body weight (BW), heart weight (HW), and tibia length (TL) were recorded. The relative heart weight was determined by calculating the HW/TL ratio.

### 2.4. Glucose and Fructosamine Levels and Lipid Profile

Blood plasma was collected to measure the glucose and fructosamine levels and lipid profile at the end of the treatment. Commercially available kits were used to measure the level of glucose (EIAGLUC, Thermo Fisher, Frederick, MD, USA), fructosamine (LS-F53786, LS Bio, Newark, CA, USA), and total cholesterol (CHOL), triglyceride (TG), LDL, and HDL (ab242305, Abcam, Cambridge, UK).

### 2.5. Thiobarbituric Acid Reactive Substances (TBARS) and Conjugated Diene (CD) Concentrations

TBARS concentration was determined according to a previously described protocol [28]. Briefly, 1 mL of 10% heart tissue homogenate (prepared in 1.15% KCl with 0.01 mol/L phosphate buffer, pH 7.4) was mixed with 2 mL of 7.5% trichloroacetic acid. The mixture was centrifuged at 1000× *g* for 10 min, and 1 mL of the resulting supernatant was combined with 0.5 mL of 0.7% 2-thiobarbituric acid. The reaction mixture was incubated for 10 min and subsequently cooled. TBARS levels were quantified at a wavelength of 532 nm using a NanoDrop 2000c UV-Vis spectrophotometer (Thermo Fisher Scientific, Waltham, MA, USA). Results were calculated using an extinction coefficient of 156,000 mol⁻^1^·L·cm⁻^1^.

To determine conjugated diene (CD) concentrations, heart tissue was homogenized in a solution containing 15 mmol/dm^3^ EDTA and 4% NaCl, as described previously [29]. In brief, lipid extraction was performed using a 1:1 mixture of chloroform and methanol. The chloroform layer was evaporated under a nitrogen atmosphere, and the residue was dissolved in cyclohexane. CD concentrations were measured spectrophotometrically at a wavelength of 233 nm using the NanoDrop 2000c UV-Vis spectrophotometer (Thermo Fisher Scientific, Waltham, MA, USA).

### 2.6. Western Blot Analysis

Heart tissue samples were homogenized, and Western blot analysis was conducted following a previously described protocol [29]. Briefly, membranes were incubated overnight at 4 °C with the following primary antibodies: anti-eNOS (1:1000, Abcam, ab5589, Cambridge, UK), anti-phospho-eNOS (1:1000, Invitrogen, #PA5-35879, Waltham, MA, USA), anti-NADPH oxidase 4 (1:2000, Abcam, ab154244), anti-NF-κB p65 (1:1000, Cell Signaling, 6956), and anti-GAPDH (1:5000, Abcam, ab201822) as a loading control. Subsequently, the membranes were incubated for 2 h at room temperature with a peroxidase-conjugated secondary goat anti-rabbit antibody (1:5000, Abcam, ab97051).

Protein bands were visualized using an enhanced chemiluminescence system (ECL, Bio-Rad, CA, USA) and quantified with a ChemiDoc™ Touch Imaging System (Image Lab™ Touch software, Bio-Rad, Hercules, CA, USA). Band intensities were normalized to GAPDH as a loading control for heart tissue samples.

### 2.7. Total NO Synthase (NOS) Activity

Total nitric oxide synthase (NOS) activity was quantified in crude heart homogenates by measuring the conversion of [^3^H]-L-arginine to [^3^H]-L-citrulline (ARC, Saint Louis, MO, USA), as described previously [30,31]. In brief, 50 µL of 20% tissue homogenate was incubated in a reaction mixture containing 0.5 M Tris-HCl (pH 7.4), 10 mM NADPH, 20 mM CaCl_2_, 100 µM [^3^H]-L-arginine, 1 mg/mL calmodulin, a 1:1 mixture of FAD and FMN, and 50 mM tetrahydrobiopterin (BH_4_), in a final volume of 100 µL. The incubation was conducted at 37 °C for 30 min. The reaction was terminated by adding 1 mL of 0.02 M HEPES buffer (pH 5.5) containing 2 mM EDTA, 2 mM EGTA, and 1 mM L-citrulline. The reaction mixture was subsequently applied to 1 mL Dowex 50WX-8 columns (Na⁺ form) to separate [^3^H]-L-citrulline. [^3^H]-L-citrulline was measured using a Quanta Smart TriCarb Liquid Scintillation Analyzer (Packard Instrument Company, Meriden, CT, USA).

### 2.8. Statistical Analysis

Data are expressed as means ± standard error of the mean (SEM). Statistical analyses were performed using one-way analysis of variance (ANOVA) followed by Bonferroni post hoc testing. Statistical significance was defined as *p* < 0.05 for both ANOVA and Bonferroni tests. Adjustments for multiple comparisons were applied to the *p*-values.

## 3. Results

### 3.1. Weight Parameters, Blood Pressure, and Glucose and Fructosamine Levels

Evolocumab treatment did not reduce body weight, which was significantly higher in obese Zucker rats compared to lean controls. There were no significant differences in heart weight, relative heart weight, and systolic blood pressure between lean and obese Zucker rats. Evolocumab treatment did not affect these parameters (Table 1). Similarly, there were no significant differences in glucose and fructosamine levels between lean and obese Zucker rats, and evolocumab treatment did not affect these parameters (Table 1).

### 3.2. Lipid Profile

**Figure 1:**
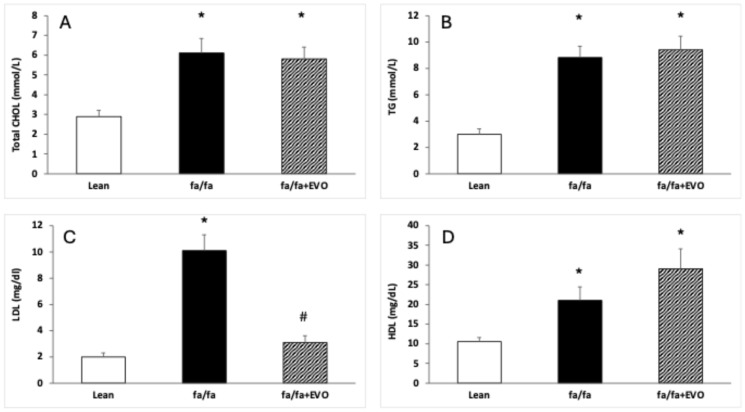
Lipid profile in the hearts of lean and obese Zucker rats (fa/fa) and obese rats treated with evolocumab (fa/fa+EVO). Total cholesterol (CHOL) level (**A**); triglyceride (TG) level (**B**); low-density lipoprotein (LDL) level (**C**); high-density lipoprotein (HDL) level (**D**). Data are expressed as means ± SEM from six animals in each group. * *p* < 0.05 compared to the lean control group; # *p* < 0.05 compared to obese Zucker rats (fa/fa).

CHOL, TG, LDL, and HDL levels were significantly increased in obese Zucker rats. Evolocumab treatment significantly decreased LDL levels but did not affect CHOL, TG, and HDL levels in obese Zucker rats (Figure 1A–D).

### 3.3. TBARS and Conjugated Diene Concentrations

**Figure 2:**
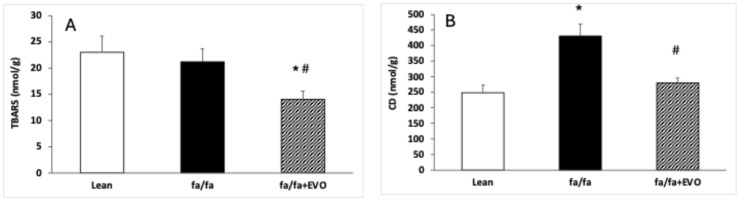
Thiobarbituric acid reactive substance (TBARS) concentration (**A**) and conjugated diene (CD) concentration (**B**) in the heart of lean and obese Zucker rats (fa/fa) and obese rats treated with evolocumab (fa/fa+EVO). Data are expressed as means ± SEM from six animals in each group. * *p* < 0.05 compared to the lean control group; # *p* < 0.05 compared to obese Zucker rats (fa/fa).

There were no significant differences in TBARS between lean and obese Zucker rats, and evolocumab treatment decreased TBARS compared to both control and obese rats (Figure 2A). CD concentration was significantly increased in obese Zucker rats, and evolocumab treatment decreased it to the level of lean controls (Figure 2B).

### 3.4. NADPH Oxidase and NF-κB Protein Expression

**Figure 3:**
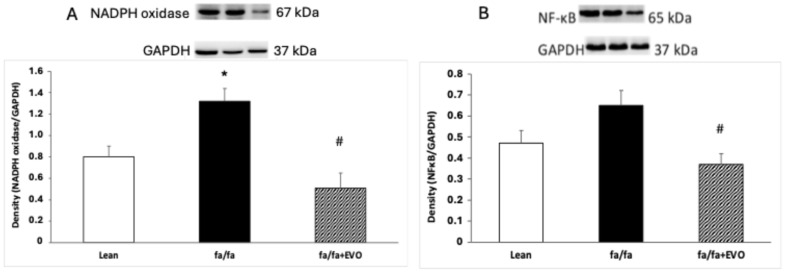
NADPH oxidase protein expression (**A**) and nuclear factor kappa B (NF-κB) protein expression (**B**) in the hearts of lean and obese Zucker rats (fa/fa) and obese rats treated with evolocumab (fa/fa+EVO). Data are expressed as means ± SEM from six animals in each group. * *p* < 0.05 compared to the lean control group; # *p* < 0.01 compared to obese Zucker rats (fa/fa).

NADPH oxidase protein expression was significantly increased in obese Zucker rats, and evolocumab treatment significantly decreased it (Figure 3A). There were no significant differences in NF-κB protein expression between lean and obese Zucker rats despite an increasing trend in obese rats. Evolocumab treatment significantly decreased NF-kappaB protein expression compared to obese Zucker rats (Figure 3B).

### 3.5. Endothelial NOS (eNOS) and Phosphorylated eNOS (peNOS) Protein Expression and Total NOS Activity

**Figure 4:**
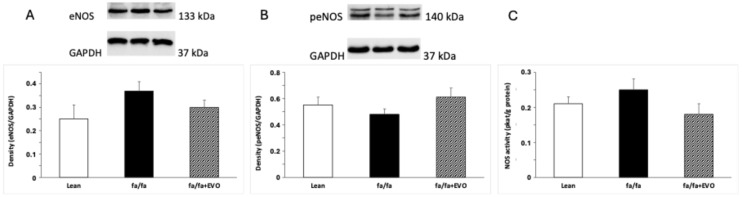
Endothelial nitric oxide synthase (eNOS) protein expression (**A**), phosphorylated eNOS (peNOS) protein expression (**B**), and total NOS activity (**C**) in the hearts of lean and obese Zucker rats (fa/fa) and obese rats treated with evolocumab (fa/fa+EVO). Data are expressed as means ± SEM from six animals in each group.

There were no significant differences in eNOS and peNOS protein expression between lean and obese Zucker rats. Evolocumab treatment did not affect these parameters significantly (Figure 4A,B), documenting any effect of evolocumab on the eNOS/peNOS pathway in the hearts of these animals. Finally, no differences in total NOS activity were observed in lean Zucker rats, obese Zucker rats, and obese Zucker rats treated with evolocumab (Figure 4C).

## 4. Discussion

Given the significant correlation between hyperlipidemia and cardiovascular disease, lowering LDL-c clearly contributes to reducing the risk of cardiovascular disease. PCSK9 inhibitors represent a promising tool for lowering this undesirable lipid while not exhibiting adverse effects like statins [32,33]. Recently, evolocumab and alirocumab have been approved by the US Food and Drug Administration to inhibit PCSK9. Although the PCSK9 inhibitor evolocumab is a human monoclonal immunoglobulin G2 (IgG2), its antioxidant, anti-inflammatory, antilipotoxic, and autophagy-suppressing effects have been demonstrated in Sprague–Dawley and transgenic rats and knockout mice [21,22,34]. In our experimental conditions, the obese Zucker rats with hyperlipidemia were used to study the effects of evolocumab on lipid profile, lipid peroxidation, proinflammatory factor NF-κB, and NO production. Because correlation analysis using wild-type C57BL6/J mice and PCSK9^−/−^ knockout mice showed that higher PCSK9 expression indicated worse cardiac function after acute myocardial infarction and that PCSK9 knockout improved cardiac function and reduced infarct size along with attenuated inflammation in this tissue [35], the heart was selected for molecular analysis in our study.

Our experimental study confirmed increased total cholesterol, triglyceride, and LDL levels in the plasma of obese Zucker rats. On the other hand, administration of the PSCK9 inhibitor evolocumab significantly decreased plasma LDL levels but had no effect on total cholesterol, triglyceride, and HDL levels. Plasma glucose and fructosamine levels were also not affected by PCSK9 inhibitor treatment. Recently, it has been shown that the use of evolocumab reduces circulating LDL-c by approximately 40–65% when used in combination with statins [36,37]. In our experiment using obese Zucker rats, LDL was reduced by 63% at the end of the 6-week treatment with evolocumab alone. Similarly, a significantly lower level of LDL (by 50%) was found in high cardiovascular risk patients after 3 months of evolocumab monotherapy [38]. In patients with familial hypercholesterolemia, evolocumab monotherapy decreased LDL levels by 58% [39]. This means that in obese Zucker rats, evolocumab reduced LDL levels comparable to human studies. In individuals with type 2 diabetes and dyslipidemia and on a maximum-tolerated statin, evolocumab decreased LDL-c by 54.3% after 12 weeks of treatment. Evolocumab had no effect on glycemic variables in this study [40]. Additionally, a systematic review and meta-analysis of metabolic and cardiovascular outcomes in patients with diabetes confirmed that PCSK9 inhibitors did not affect glucose metabolism [41]. Similarly, in our experimental conditions, no significant effect of evolocumab on glucose or fructosamine levels was observed.

However, phase-3 interventional studies have demonstrated that beyond the lipid-lowering effects of PCSK9 inhibitors, including evolocumab, they exhibit pleiotropic effects. Their pleiotropic effects mainly include antithrombotic, antiatherosclerotic, and anti-inflammatory abilities, which may lie in the antioxidant properties of these PCSK9 inhibitors [36,37]. Indeed, in our experimental study, evolocumab treatment decreased NADPH oxidase activity and reduced the TBARS concentration and lipid peroxidation, as documented by a decreased CD level in the heart. In a myocardial ischemia–reperfusion (I/R) model in Wistar rats, a PCSK9 inhibitor administered before ischemia exerted a cardioprotection, as demonstrated by the attenuation of infarct size and cardiac arrhythmia during cardiac I/R injury. The authors documented that the attenuation was associated with decreased mitochondrial ROS production [42]. Additionally, Ding et al. [43] provided evidence that low experimental shear stress increased PCSK9 expression in association with ROS production in vascular endothelial and smooth muscle cells. The authors hypothesized that ROS may regulate PCSK9 expression and the development of atherosclerosis in arterial channels with low shear stress [43]. Similarly, Safaeian et al. [44] showed that pretreatment of human umbilical vein endothelial cells with evolocumab reduced hydroperoxide and malondialdehyde levels when stimulated with hydrogen peroxide at different concentration ranges. In a clinical study, Lankin et al. [45] reported the effect of evolocumab on reducing the plasma concentration of ox-LDL in patients with coronary artery diseases without affecting the activity of antioxidant enzymes [45]. In our study, evolocumab reduced NADPH oxidase activity, potentially leading to reduced superoxide production and decreased LDL oxidation. It is likely that evolocumab may contribute to the reduction of the atherosclerotic process in this way. Similarly, Yang et al. 2023 [46] reported that evolocumab treatment reduced oxidative stress, lipid deposition, and plaque lesion area in apolipoprotein E−/− mice fed a high-fat diet [46].

Recently, PCSK9-induced NF-κB stimulation has been identified in hypoxia/reoxygenation stress in primary murine cardiomyocytes [47]. Moreover, Shin et al. [48] reported that PCSK9 activates tyrosine kinase, protein kinase C delta, and NF-κB, leading to the progression of the atherosclerotic process independently of LDL receptors [48]. In our experimental study, evolocumab treatment reduced NF-κB expression in the heart of obese Zucker rats, which could also be a consequence of reduced superoxide production. Similar results have been shown by Lei et al. [49]. The authors documented that PCSK9 inhibition suppressed the activation of TLR4/NF-κB in a rat model of colitis [49]. Additionally, in the brain of Wistar rats fed a high-fat-cholesterol diet, a PCSK9 inhibitor decreased different inflammatory mediators, including NF-κB [50]. Luo et. al. even speculated that the reduction in thrombocyte and cell adhesive molecules after evolocumab treatment in a mouse model of ischemic stroke was mediated by the inhibition of the ERK/NF-κB pathway [51].

In our study, we clearly showed that evolocumab, in addition to its lipid-lowering effect, also had a positive effect on the consequences caused by increased LDL cholesterol levels. Evolocumab was able to reduce oxidative stress and lipid peroxidation, which was accompanied by a decreased expression of pro-inflammatory factor NF-κB. The evidence of this beneficial effect of evolocumab in the heart is among the strengths of our experimental study.

In the vascular wall, vascular smooth muscle cells are the main PCSK9-secreting cells. However, many studies have documented that endothelial cells also express PCSK9, albeit to a lesser extent than smooth muscle cells [43]. PCSK9 has been found to promote endothelial dysfunction during sepsis [52]. Furthermore, in diabetes, PCSK9 suppressed the expression and activation of protein kinase B (AKT) and eNOS in endothelial cells [53]. On the other hand, the addition of evolocumab on top of empagliflozin improved endothelial function in individuals with type 2 diabetes [54]. These results may indicate the involvement of eNOS expression and eNOS phosphorylation in the improvement of endothelial function. Thus, in our experimental study, protein expression of cardiac eNOS and peNOS was measured as well. Using obese Zucker rats, however, we did not observe any significant changes in cardiac eNOS and peNOS protein expression or total NOS activity after evolocumab treatment. In terms of the atherosclerotic process, it would be appropriate to analyze the impact of evolocumab on the vascular NO signaling pathway, especially on endothelial and inducible NOS, which would have a greater informative value in relation to atherosclerosis. This is a limitation of our study. The study of Zulkapli et al. [55] showed ambiguous results in the upregulation of eNOS gene or protein expression in stimulated human coronary artery endothelial cells after evolocumab treatment [55]. Thus, further studies are needed to clarify the effect of PCSK9 inhibitors on the NO pathway.

## 5. Conclusions

With the increasing global prevalence of obesity, it is necessary to determine the factors and signaling pathways that may underlie obesity-related cardiac dysfunction. Concurrently, it is important to search for an effective treatment that, in addition to safely adjusting the lipid profile, can positively influence cardiac signaling pathways.

We demonstrated that, in addition to its lipid-lowering ability, evolocumab treatment reduced the ROS production and lipid peroxidation associated with decreased expression of the inflammatory factor NF-κB in the heart of obese Zucker rats. However, no changes were observed in eNOS expression or phosphorylation or in total NOS activity, indicating that evolocumab did not affect the cardiac NO signaling pathway.

However, the reduction of ROS generation in the heart of obese Zucker rats may indicate that evolocumab was also able to reduce the consequences of elevated LDL cholesterol levels, namely oxidative stress and lipid peroxidation. These are common causes of endothelial dysfunction and myocardial ischemia. Decreased expression of the pro-inflammatory factor NF-κB further suggested that a reduction in oxidative stress and lipid peroxidation was followed by a reduced inflammatory process. Evolocumab could thus be among the effective drugs that can reduce the consequences of elevated LDL cholesterol, especially in obesity-related heart disease.

## Figures and Tables

**Table 1:** Body weight (BW), heart weight (HW), relative heart weight as HW/tibia length (TL), blood pressure (BP), and glucose and fructosamine levels in the heart of lean and obese Zucker rats (fa/fa) and obese rats treated with evolocumab (fa/fa+EVO).

	BW [g]	HW [g]	HW/TL [g/cm]	BP [mmHg]	Glucose [mmol/L]	Fructosamine [μmol/L]
**lean**	383 ± 27	1.21 ± 0.09	0.325 ± 0.021	132 ± 4	8.0 ± 0.9	177 ± 5
**fa/fa**	521 ± 3 2 **	1.22 ± 0.03	0.351 ± 0.003	133 ± 5	7.7 ± 0.5	190 ± 8
**fa/fa+EVO**	508 ± 29 **	1.19 ± 0.05	0.352 ± 0.015	119 ± 8	6.9 ± 0.4	191 ± 8

Data are expressed as means ± SEM from six animals in each group. ** *p* < 0.01 compared to the lean control group.

## Data Availability

Data supporting the reported results can be found in the archived datasets of the Department of Neuro-Cardiovascular Interactions, Institute of Normal and Pathological Physiology, Center of Experimental Medicine, Slovak Academy of Sciences.
